# Physical activity and wellbeing among pre-service helping professionals: the role of lifestyle in a Hungarian university sample

**DOI:** 10.3389/fpubh.2026.1898956

**Published:** 2026-07-13

**Authors:** Erzsébet Rákó, Attila Lengyel, Zoltán Szerdahelyi, Anetta Müller

**Affiliations:** 1 Department of Social Pedagogy, Faculty of Education for Children and Special Educational Needs, University of Debrecen, Debrecen, Hungary; 2 Coordination and Research Centre for Social Sciences, Faculty of Economics and Business, University of Debrecen, Debrecen, Hungary; 3 Department of Complex Arts and Health Education, Faculty of Education for Children and Special Educational Needs, University of Debrecen, Debrecen, Hungary; 4 Institute of Sport Economics and Management, University of Debrecen, Debrecen, Hungary

**Keywords:** helping professions, lifestyle coherence, motivation, physical activity, student wellbeing, university students

## Abstract

Physical activity is widely assumed to contribute to psychological wellbeing; however, this relationship may reflect broader lifestyle patterns rather than a direct causal effect. This cross-sectional study examined physical activity levels, subjective wellbeing, and their predictors in a sample of 1,109 pre-service students enrolled in helping-profession programs (special education, infant and early childhood education, pre-school education, and social education) at the University of Debrecen, Hungary. Data were collected using an online questionnaire assessing physical activity habits, wellbeing, dietary behavior, sleep, and motivational factors. Hierarchical multiple regression models were used to analyze predictors of weekly physical activity and wellbeing. Physical activity did not independently predict wellbeing after lifestyle variables were controlled. Instead, wellbeing was predicted by daily vegetable consumption, enjoyment of university physical education, motivator count, sleep adequacy, financial situation, and attention to healthy eating. Physical activity volume was predicted by perceived importance of physical activity, work intensity, and barrier count. Part-time students who combined work and study reported higher physical activity levels and better wellbeing than full-time students. The findings suggest that wellbeing in this population is associated more strongly with coherent lifestyle patterns than with physical activity volume alone.

## 1 Introduction

Physical activity (PA) is one of the most robustly documented determinants of human health. The World Health Organization recommends that adults perform at least 150–300 min of moderate-intensity, or 75–150 min of vigorous-intensity aerobic PA per week, alongside muscle-strengthening activities on at least 2 days per week (1). Despite the strength of this evidence base, physically inactive populations remain a global public health concern, with substantial proportions of adults in high-income countries failing to meet these recommendations (2, 3). University students represent a particularly important group in this context: the transition into higher education is commonly associated with a restructuring of daily routines, reduced structured PA opportunities, and increased academic and occupational demands that can displace health-promoting behaviors (4, 5).

A growing body of evidence documents insufficient levels of PA among university students across multiple national contexts (5). Systematic reviews consistently identify lack of time and lack of motivation as the most frequently reported barriers, followed by absence of suitable facilities, financial constraints, and health-related limitations (4, 6). Facilitators of PA engagement include enjoyment, social support, self-efficacy, and perceived competence suggesting that psychological and contextual factors jointly shape whether students translate intentions into regular behavior (7). Importantly, prior physical education (PE) experiences also appear to play a formative role: students who report positive experiences in school or university PE are more likely to express PA-related motivation and confidence in adulthood (8).

Self-Determination Theory (SDT) (9) provides a well-established theoretical framework for understanding PA motivation. The theory proposes that three basic psychological needs autonomy, competence, and relatedness underlie self-motivated and psychologically healthy behavior. Motivation is conceptualized along a continuum ranging from amotivation through controlled regulation to autonomous, self-determined forms of motivation, with more autonomous forms consistently predicting greater PA engagement and longer-term adherence (10). Applied to the university student context, SDT implies that students who endorse intrinsic or identified reasons for exercising such as health maintenance, fitness, or genuine enjoyment are more likely to sustain PA over time than those motivated primarily by external pressures or social comparison (10). In the present study motivational content is approached via participants’ endorsed motivators and barriers, perceived PA importance, and evaluative attitudes towards PE.

PA has long been proposed as a key contributor to subjective wellbeing. Meta-analytic evidence indicates that PA interventions can reduce symptoms of depression and anxiety and improve overall psychological functioning in university student populations (11). However, the relationship between PA and wellbeing is more conditional than often assumed (12). It appears to be moderated by the type, intensity, and setting of activity, as well as by individual characteristics including motivation quality consistent with SDT predictions (10, 11). Critically, in population-based cross-sectional studies that simultaneously include multiple lifestyle predictors, the independent contribution of PA to wellbeing often diminishes substantially once diet quality, sleep adequacy, and socioeconomic factors are accounted for (13). This suggests that wellbeing may be less specifically tied to PA volume per se than to a broader pattern of health-supporting behaviors.

Diet quality is an important co-predictor of wellbeing that is frequently studied independently of PA. A comprehensive systematic review found consistent, if modest, associations between unhealthy dietary practices and poorer mental health across university student samples, with cross-sectional evidence pointing to the particular relevance of vegetable and fruit consumption (14). The mechanisms are thought to involve both nutritional pathways (e.g., micronutrient adequacy, gut-brain axis) and behavioral clustering, in which students with generally health-conscious lifestyles tend to score better across multiple health domains simultaneously. This clustering complicates attribution: when diet and PA covary, isolating the independent predictive value of each requires their simultaneous inclusion in regression models, a methodological consideration that is often neglected in the literature (15).

Sleep is a further component of health that intersects with both PA and wellbeing (16). Poor sleep quality is highly prevalent among university students and is robustly associated with reduced psychological wellbeing, heightened anxiety and depression symptoms, and impaired academic performance (17, 18). The relationship between sleep and wellbeing is plausibly bi-directional: poor wellbeing can disrupt sleep, while inadequate sleep in turn erodes emotional regulation capacity and daytime functioning. In multivariate models, sleep tends to emerge as one of the stronger predictors of student wellbeing, often accounting for unique variance beyond that explained by PA alone. Yet studies rarely model PA, diet, and sleep simultaneously, leaving their relative independent contributions to wellbeing unclear.

The present study examines these questions in a sample with particular professional relevance: pre-service students enrolled in helping-profession programs special education, infant and early childhood education, pre-school education, and social education at the University of Debrecen, Hungary. Graduates of these programs are expected to promote health-related behaviors, physical development, and wellbeing among the children, families, and vulnerable individuals they serve. Their own health behaviors and wellbeing therefore carry a dual significance: they are outcomes of interest in their own right, and they may also shape professional socialization into health-promoting roles. Despite this double relevance, PA and wellbeing in this specific student population have received limited empirical attention (19, 20). A further structural feature of this sample warrants attention: the majority of students (70.7%) are part-time students who are simultaneously employed. This combination of work and study is characteristic of these programs in Hungary and represents a lifestyle configuration marked by time pressure, reduced discretionary time, and adult responsibilities that differs substantially from the full-time, school-leaver norm that implicitly underlies much university student PA research.

The present study addresses three related aims. First, it describes the PA levels, wellbeing, and associated lifestyle and motivational characteristics of pre-service helping professionals, with attention to differences by program of study and study form. Second, it examines the predictors of weekly PA volume using a hierarchical regression model that sequentially enters sociodemographic, lifestyle, and motivational blocks. Third, it examines the predictors of subjective wellbeing using an analogous hierarchical model that explicitly tests the independent contribution of PA volume after lifestyle factors are controlled. In doing so, the study responds to calls for more nuanced, multivariate investigations of the determinants of PA and wellbeing in non-traditional university student populations.

## 2 Materials and methods

### 2.1 Study design and participants

This study employed a cross-sectional survey design. Data were collected from pre-service helping professionals enrolled at the University of Debrecen, Hungary, using an online questionnaire. Participation was voluntary and anonymous. By completing the questionnaire, participants consented to the use of their responses for research purposes.

The study received ethical approval (GYGYK-3/2025) from the Scientific Committee of Faculty of Education for Children and Special Educational Needs at the University of Debrecen.

The target population comprised students enrolled in four helping-profession programs: special education, infant and early childhood education, pre-school education, and social education. These programs were selected because their graduates are expected to promote health-related behaviors, physical activity, and wellbeing among children, families, and vulnerable populations making their own health profiles professionally relevant.

A total of 1,109 valid responses were obtained. Detailed sample characteristics are reported in the Results section (Table 1). The sample was predominantly female (95.6%), with a mean age of 32.0 years (SD = 10.7, range 18–67). The age distribution and high proportion of part-time students (70.7%) reflect the structure of these programs in Hungary, where most students are employed adults combining work and study, rather than school-leavers entering higher education directly.

**Table 1:** Overview of measures used in the present study.

Construct	Survey question (abbreviated)	Response format	Role in analysis	α/reliability
Gender	Gender (Q1)	Binary: woman/man	Demographic descriptor	–
Age	Numeric age in years (Q3)	Open numeric entry	Sociodem. control	–
Field of study	Which programme are you enrolled in? (Q4)	4 categories	Dummy-coded control (ref: Special Education)	–
Year of study	Which year of study are you in? (Q5)	Ordinal 1–5	Sociodem. control	–
Study form	What type of training program are you in? (Q6)	Binary: full-time = 1, part-time = 0	Sociodem. control	–
Residence	Permanent place of residence (Q7)	5 categories	Descriptive only	–
Financial situation	Financial situation (Q8)	Scale 1–10	Sociodem. control	–
Parental education	Mother’s (Q10) and father’s (Q11) highest educational attainment	Ordinal 1–4 (collapsed)	Sociodem. control	–
Work intensity	Do you work alongside your studies? (Q12)	Ordinal 1–5	Sociodem. control	–
Smoking	Do you smoke? (Q14)	Binary yes/no → 1/0	Lifestyle covariate	–
Alcohol frequency	How often do you consume alcohol? (Q15)	Ordinal 1–5	Lifestyle covariate	–
Sleep	How many hours do you sleep per day on average? (Q16)	Ordinal 1–4 (<5 h to >8 h)	Lifestyle covariate	–
Healthy eating attention	How much attention do you pay to healthy eating? (Q19)	Scale 1–5	Lifestyle covariate (Model B)	–
Eating behaviors	5 items: daily vegetables/fruit, fast food, ready meals, energy drinks (Q27)	Scale 1–6 (endpoints defined)	Individual items as covariates	α = 0.488 (index not used)
Chronic illness	Do you have a chronic illness? (Q39)	Ordinal 0–2	Lifestyle covariate	–
Weekly sport frequency	How many times per week do you do sport? (Q20)	Ordinal 0–3 (0 to ≥5 sessions)	Component of PA index (DV1)	–
PA type frequency	How often do you engage in: sport, walking, cycling, household activities, work-related activity? (Q31)	Ordinal 1–5 (almost never to daily)	Sport item: component of PA index (DV1)	–
Weekly PA minutes	How many minutes per week do you spend on physical activity on average? (Q32)	Ordinal 1–4 (0–60 to >300 min)	Primary DV1	–
Exercise type	What type of physical exercise do you do? (Q33)	Categorical (7 options + open)	Descriptive only	–
Exercise environment	In what kind of environment do you usually exercise? (Q34)	Categorical (4 options)	Descriptive only	–
Wellbeing index	5 items: cheerful, calm, active, fresh on waking, interesting days (Q21)	Scale 1–4 per item; mean composite	DV2	α = 0.808
Motivators (count)	Health, fitness, body, social, competition -endorsed motivators (Q35)	Binary yes/no per item; sum 0–5	Motivation predictor	–
Barriers (count)	Time, motivation, health, money, facilities -endorsed barriers (Q36)	Binary yes/no per item; sum 0–5	Motivation predictor	–
PA importance	How important is regular physical exercise to you? (Q37)	Scale 1–10	Motivation predictor	–
PE enjoyment -secondary	How much did you enjoy PE classes in secondary school? (Q29)	Scale 1–10	Motivation predictor	–
PE enjoyment -university	How much do you enjoy university PE courses? (Q30)	Scale 1–10	Motivation predictor	–

Q-numbers refer to the original survey question order. DV1, first dependent variable (physical activity volume); DV2, second dependent variable (wellbeing). α = Cronbach’s alpha.

### 2.2 Measures

The survey instrument was developed by the research team at the University of Debrecen to assess physical activity habits, wellbeing, and related behaviors among pre-service helping professionals. All items were presented in Hungarian. The variables used in the present study are listed in Table 1, together with the corresponding survey question, response format, and role in the analysis. Variables not included in the present article are reserved for separate publications drawing on the same dataset (Table 1).

#### 2.2.1 Sociodemographic variables

Sex (Q1) was recorded as a binary item (female/male). Age was recorded both categorically (Q2) and as a numeric open entry (Q3); only the numeric version was used in analyses. Field of study (Q4) comprised four categories and was dummy-coded for regression, with special education as the reference category. Year of study (Q5) was treated as an ordinal variable (1–5). Study form (Q6) was dichotomized: full-time students were coded 1 and part-time students 0. Permanent residence (Q7) was recorded across five settlement types and is reported descriptively.

Socioeconomic status was captured through three indicators: self-reported financial situation (Q8), rated on a scale from 1 (very poor circumstances) to 10 (very good circumstances); mother’s highest educational attainment (Q10); and father’s highest educational attainment (Q11). Both parental education variables were originally recorded across five categories and recoded to an ordinal scale from 1 (primary education or less) to 4 (university or college degree); responses in the residual category “other” were treated as missing. Work intensity alongside study (Q12) was recorded across five categories from “does not work” (1) to “works multiple days per week, more than 4 h per day” (5).

#### 2.2.2 Health and lifestyle variables

Smoking (Q14) was recorded as a binary yes/no item and coded 1 (smoker) or 0. Alcohol consumption frequency (Q15) was recorded on a five-point ordinal scale from “never” (1) to “daily” (5). Sleep duration (Q16) was recorded in four ordinal categories: fewer than 5 h (1), 5–6 h (2), 7–8 h (3), and more than 8 h (4).

Attention to healthy eating (Q19) was rated on a scale from 1 (not at all) to 5 (completely). Specific eating behaviors (Q27) were assessed using five items rated on a 1–6 scale with defined endpoints (1 = not at all characteristic; 6 = completely characteristic): daily vegetable consumption, daily fruit consumption, fast food consumption, ready meal consumption, and energy drink consumption. Cronbach’s alpha for these five items was 488, indicating insufficient internal consistency for composite use. Accordingly, individual items were entered separately in regression analyses rather than as an index. In Model B, only daily vegetable consumption and the Q19 healthy eating attention item were retained as predictors, following inspection of bivariate relationships and a variance inflation factor check that identified multicollinearity between the fruit and vegetable items (inter-item *r* = 74) and suppressor effects for the unhealthy eating items when entered simultaneously (see also section 2.4). The remaining Q27 items are reported descriptively.

Presence of chronic illness (Q39) was assessed with a three-category item: no chronic illness (0), chronic illness not requiring regular medication (1), and chronic illness requiring regular medication (2), and was treated as an ordinal variable in analyses.

#### 2.2.3 Physical activity variables

Physical activity was assessed through multiple complementary items. Weekly sport session frequency (Q20) was recorded in four categories (0, 1–2, 3–4, or 5 or more sessions per week) and recoded to an ordinal scale of 0–3. Frequency of specific physical activity types (Q31) was assessed across five activities (sport, walking, cycling, household-related PA, work-related PA) on a five-point scale from “almost never” (1) to “daily” (5). Weekly PA volume (Q32) was assessed in four ordinal categories: 0–60 min (1), 61–150 min (2), 151–300 min (3), and more than 300 min (4), and served as the primary dependent variable in Model A.

A PA frequency index was constructed as the mean of the recoded weekly sport frequency (Q20) and the sport item from Q31, providing a more stable estimate of overall sport-specific activity frequency. This index served as a supplementary PA measure in Model B. Exercise type (Q33) and exercise environment (Q34) were recorded categorically and are reported descriptively.

#### 2.2.4 Wellbeing

Subjective wellbeing was assessed using five items adapted from the WHO-5 WellBeing Index (Q21). Participants rated the extent to which, over the previous 2 weeks, they had felt: cheerful and in good spirits; calm and relaxed; active and vigorous; woke up feeling fresh and rested; and experienced daily life as full of interesting things. Each item was rated on a four-point scale (1 = not at all characteristic; 4 = completely characteristic). A wellbeing index was computed as the mean of the five items. Internal consistency was acceptable (Cronbach’s *α* = 0.808).

It should be noted that the original WHO-5 uses a six-point response scale (0–5); the present adaptation uses a four-point scale, and the instrument is therefore referred to as a wellbeing index rather than the WHO-5 throughout this article. Because of this modification, the resulting scores are not directly comparable with those obtained from the validated WHO-5, and the adapted version was not subjected to separate validation beyond the internal-consistency analysis reported above; the index should therefore be interpreted as a study-specific measure of subjective wellbeing rather than as a standardized WHO-5 score.

#### 2.2.5 Motivation and attitude variables

Motivators for physical activity (Q35) were assessed by asking participants to endorse (yes/no) each of five potential motivations: health maintenance, fitness and wellbeing, body improvement, social reasons (friends/company), and competition/performance. A motivator count was computed as the sum of endorsed items (range 0–5). Barriers to regular physical activity (Q36) were assessed analogously with five items: time pressure, lack of motivation/willingness, health reasons, financial constraints, and absence of suitable facilities. A barrier count was computed as the sum of endorsed barriers (range 0–5).

Perceived importance of regular exercise for health (Q37) was rated on a scale from 1 (not at all important) to 10 (completely important). Enjoyment of secondary school physical education (Q29) and university compulsory PE courses (Q30) were each rated on a 1–10 scale (1 = did not enjoy at all; 10 = enjoyed very much) and served as attitudinal predictors of both PA and wellbeing.

### 2.3 Data preparation

Numeric age entries (Q3) were cleaned prior to analysis: three text responses were converted to their numeric equivalents, and two entries with obvious typographical errors were corrected to plausible values consistent with the corresponding categorical response in Q2. No missing values remained for age after cleaning.

All ordinal and categorical variables were recoded to numeric format as described above. Dummy variables were created for field of study (reference: special education) and entered as a block in all regression models. Residence was examined as a predictor in preliminary regression models but generated variance inflation factors (VIF) exceeding 10 for three of its four dummy categories, attributable to the very small reference group (capital city residents, *n* = 21). Residence was therefore retained for descriptive purposes only and excluded from regression analyses.

Composite indices were computed as follows. The wellbeing index was the arithmetic mean of the five Q21 items. The PA frequency index was the mean of recoded Q20 (weekly sport sessions) and the sport frequency item from Q31. The motivator count and barrier count were the sums of endorsed yes-responses to Q35 and Q36, respectively.

Listwise deletion was applied to missing data in regression analyses. Missing data arose only from the parental education items (Q10 and Q11), where respondents selecting “other” were treated as missing (*n* = 23, 2.1%). The analytic sample for regression models was therefore *N* = 1,086.

### 2.4 Statistical analysis

All analyses were conducted in Python (version 3.12) using the pandas, scipy, numpy, and statsmodels libraries. Descriptive statistics were computed for all variables. Normality was assessed using the Shapiro–Wilk test on a random subsample of *n* = 500. As all continuous variables deviated significantly from normality (all *p* < 0.001), Spearman rank correlations were used for bivariate association analyses, and Mann–Whitney U tests and Kruskal-Wallis H tests were used for group comparisons. Effect sizes for Mann–Whitney tests are reported as r = 1 − (2 U)/(n₁n₂). *Post-hoc* pairwise comparisons following significant Kruskal-Wallis tests were conducted using Mann–Whitney U with Bonferroni correction for six comparisons.

Two hierarchical multiple regression models were estimated using ordinary least squares. Although both dependent variables were ordinal, OLS regression was retained to permit hierarchical entry of predictor blocks, the estimation of comparable standardized coefficients, and incremental variance (ΔR^2^) decomposition across steps; OLS estimates are generally robust for ordered outcomes with four or more categories. To confirm that this choice did not distort the substantive conclusions, the final model for weekly PA volume was additionally estimated using ordinal logistic (proportional-odds) regression as a sensitivity analysis (see section 3.4). Model A examined predictors of weekly PA volume (Q32, primary dependent variable). Variables were entered in three steps: (1) sociodemographic controls (age, year of study, study form, financial situation, mother’s and father’s education, work intensity, field of study dummies); (2) health and lifestyle covariates (smoking, alcohol, sleep, healthy eating attention Q19, five individual Q27 eating behavior items, chronic illness); and (3) motivation and attitude variables (motivator count, barrier count, PA importance Q37, secondary school PE enjoyment Q29, university PE enjoyment Q30).

Model B examined predictors of the wellbeing index (Q21, second dependent variable). Variables were entered in four steps: (1) sociodemographic controls (as in Model A); (2) health and lifestyle covariates a reduced set retaining only sleep (Q16), healthy eating attention (Q19), daily vegetable consumption from Q27, and chronic illness (Q39), following inspection of bivariate correlations and a VIF diagnostic that identified suppressor effects among the Q27 eating items when all five were entered simultaneously; (3) PA variables (PA minutes per week Q32 and PA frequency index); and (4) motivation and attitude variables (as in Model A).

For each step, R^2^, adjusted R^2^, and F-change statistics are reported. In the final model of each regression, standardized beta (*β*) coefficients are also reported to allow comparison of predictor magnitudes across variables with different scales. VIF values were inspected for all final models; no remaining predictor exceeded a VIF of 2.6, indicating acceptable multicollinearity. The significance threshold was set at *α* = 0.05 for all tests.

## 3 Results

### 3.1 Sample characteristics

The final sample comprised 1,109 pre-service helping professionals enrolled at the University of Debrecen (Hungary). The sample was predominantly female (*n* = 1,060, 95.6%), with 49 men (4.4%), reflecting the heavily feminized composition of these programs in Hungary. Participants ranged in age from 18 to 67 years (*M* = 32.0, SD = 10.7). The largest group studied special education, 34.3%, followed by infant and early childhood education, 27.1%, pre-school education, 25.6%, and social education, 13.1%. The majority were enrolled in part-time programs (70.7%), and most were in their first 3 years of study (97.9%). The full sample description is presented in Table 2.

**Table 2:** Sample characteristics (*N* = 1,109).

Variable	*n*	%
Gender		
Women	1,060	95.6
Men	49	4.4
Field of study		
Special education	380	34.3
Infant and early childhood educator	300	27.1
Preschool teacher	284	25.6
Social pedagogy	145	13.1
Study form		
Part-time	784	70.7
Full-time	325	29.3
Year of study		
1st year	402	36.2
2nd year	348	31.4
3rd year	336	30.3
4th–5th year	23	2.1
Permanent residence		
Town	526	47.4
Village/rural	323	29.1
County seat	236	21.3
Capital city/farmstead	24	2.2
Mother’s education		
Primary or less	130	11.7
Vocational	368	33.2
Secondary	387	34.9
University/college	213	19.2
Work alongside study		
Does not work	220	19.8
Occasionally	125	11.3
1–2 days/week	31	2.8
Multiple days, ≤4 h/day	46	4.1
Multiple days, >4 h/day	687	61.9

n, frequency; %, valid percentage.

In terms of socioeconomic background, the mean self-reported financial situation was *M* = 6.67 (SD = 1.47) on a 1–10 scale. The largest parental education group was secondary school graduation for mothers (34.9%) and vocational training for fathers (56.1%). The majority of participants (61.9%) worked more than 4 h per day alongside their studies, which is consistent with the dominance of part-time enrolment and the older average age of the sample.

### 3.2 Descriptive statistics for key variables

Descriptive statistics for continuous and key composite variables are presented in Table 3. Reliability analysis indicated acceptable internal consistency for the wellbeing index (Cronbach’s *α* = 0.808, five items). The healthy eating index based on Q27 items yielded insufficient internal consistency (α = 0.488) and was therefore not used as a composite; items were instead examined individually or as the single Q19 item in regression analyses.

**Table 3:** Descriptive statistics for continuous variables.

Variable	*M*	SD	Min	Max
Sociodemographic				
Age (years)	32.0	10.7	18	67
Financial situation (1–10)	6.67	1.47	1	10
Health and lifestyle				
Healthy eating attention (1–5)	3.25	0.79	1	5
Daily vegetables (1–6)	4.07	1.43	1	6
Daily fruit (1–6)	4.05	1.41	1	6
Fast food consumption (1–6)	2.27	1.05	1	6
Ready meal consumption (1–6)	2.55	1.38	1	6
Energy drink consumption (1–6)	1.95	1.55	1	6
Physical activity				
PA frequency index (0.5–4.0)	1.65	0.84	0.5	4.0
PA importance (1–10)	7.31	2.05	1	10
PE enjoyment -secondary school (1–10)	6.50	2.92	1	10
PE enjoyment -university (1–10)	6.65	2.83	1	10
Motivator count (0–5)	3.26	1.04	0	5
Barrier count (0–5)	2.13	1.10	0	5
Wellbeing				
Wellbeing index (1–4)	2.71	0.56	1.0	4.0
Cheerful and good-humored	3.18	0.65	1	4
Calm and relaxed	2.60	0.78	1	4
Active and vigorous	2.77	0.73	1	4
Woke up fresh and rested	2.23	0.84	1	4
Daily life full of interesting things	2.77	0.74	1	4

Wellbeing index: mean of five items, 1 = not at all characteristic, 4 = completely characteristic (α = 0.808). PA frequency index: composite of weekly sport frequency (Q20) and sport activity frequency from Q31, range 0.5–4.0. Motivator count and barrier count: sum of endorsed items from Q35 and Q36, respectively, (range 0–5).

Physical inactivity was widespread in the sample. Nearly half of respondents (44.7%) reported engaging in no structured sport activity in a typical week, and a further 43.7% reported only 1–2 sessions per week. Only 11.5% reported three or more weekly sport sessions. In terms of total weekly PA volume, 21.9% reported fewer than 60 min per week, while 16.0% reported more than 300 min. The most frequently reported leisure-time exercise type was walking (53.2%), followed by cycling (17.0%), strength training/fitness (12.8%), yoga/pilates (5.7%), running (4.8%), and ball sports (2.8%). The preferred exercise environment was outdoors (62.8%), followed by home (22.8%) and a gym or sports facility (11.7%). A small proportion (2.6%) indicated they do not exercise at all.

Despite widespread inactivity, participants rated the importance of regular exercise highly (*M* = 7.31, SD = 2.05). The most commonly endorsed motivators were health maintenance (90.2%), fitness and wellbeing (87.8%), and body improvement (84.2%). Social motives (50.5%) and competition (13.7%) were less prevalent. Time pressure was the dominant barrier, reported by 87.6% of participants, followed by lack of motivation (58.3%), absence of suitable facilities (28.4%), health reasons (21.8%), and financial constraints (17.1%).

Mean wellbeing index score was *M* = 2.71 (SD = 0.56) on a 1–4 scale. Among the five items, participants scored highest on feeling cheerful and good-humored (*M* = 3.18) and lowest on waking up fresh and rested (*M* = 2.23), suggesting particular difficulty with sleep-related wellbeing.

Regarding sleep, 47.1% of participants reported sleeping only 5–6 h on average, and a further 6.5% reported fewer than 5 h. Only 43.8% reported the recommended 7–8 h. Smoking was reported by 27.1% of participants. Alcohol consumption was moderate: 36.6% reported never drinking, 56.7% reported 1–2 times per month, and only 6.7% reported weekly or more frequent consumption. A chronic illness was reported by 27.8% of the sample, with 16.8% requiring regular medication.

### 3.3 Group differences by field of study and study form

Kruskal-Wallis tests were conducted to examine differences across fields of study in key outcome and predictor variables (Table 4). No significant differences were found across fields in PA frequency [*H*(3) = 1.08, *p* = 0.782], PA volume [*H*(3) = 4.26, *p* = 0.235], PA importance [*H*(3) = 3.55, *p* = 0.314], motivator count [*H*(3) = 4.75, *p* = 0.191], or barrier count [*H*(3) = 4.81, *p* = 0.186]. Physical activity behavior was thus homogeneous across program areas.

**Table 4:** Group differences by field of study: key variables.

Variable	Special education M(SD)	Infant and early childhood educator M(SD)	Preschool teacher M(SD)	Social pedagogy M(SD)	H (p)
Wellbeing index†	2.61 (0.60)	2.79 (0.55)	2.78 (0.53)	2.67 (0.52)	22.34***
PA frequency index	1.63 (0.82)	1.69 (0.87)	1.62 (0.84)	1.64 (0.86)	1.08 (0.78)
PA minutes/week	2.26 (0.93)	2.40 (1.05)	2.36 (1.00)	2.43 (1.01)	4.26 (0.23)
PA importance	7.39 (2.01)	7.22 (2.12)	7.41 (2.00)	7.09 (2.06)	3.55 (0.31)
Motivator count	3.21 (1.00)	3.32 (1.13)	3.35 (0.95)	3.12 (1.09)	4.75 (0.19)
Barrier count	2.23 (1.11)	2.12 (1.14)	2.05 (1.05)	2.06 (1.06)	4.81 (0.19)
PE enjoyment -university†	6.10 (2.84)	6.48 (2.82)	7.71 (2.39)	6.37 (3.06)	55.83***
Age (years)	29.97 (10.79)	29.87 (10.87)	35.52 (8.60)	34.99 (11.73)	81.10***
Work intensity	3.37 (1.76)	3.63 (1.73)	4.29 (1.43)	4.08 (1.55)	55.05***

H = Kruskal-Wallis test statistic. †Significant *post-hoc* pairwise differences: Wellbeing—Special Education < Infant and Early Childhood Educator (*p* = 0.0004, *r* = 0.18) and Special Education < Preschool Teacher (*p* = 0.0007, *r* = 0.17). PE enjoyment (university)—Preschool Teacher > Special Education (*p* < 0.001, *r* = 0.33), Preschool Teacher > Infant and Early Childhood Educator (*p* < 0.001, *r* = 0.25), Preschool Teacher > Social Pedagogy (*p* < 0.001, *r* = 0.25). *p < 0.05. **p < 0.01. ***p < 0.001.

By contrast, wellbeing differed significantly across fields [*H*(3) = 22.34, *p* < 0.001]. *Post-hoc* pairwise Mann–Whitney tests with Bonferroni correction revealed that special education students reported significantly lower wellbeing than both infant and early childhood educator students (*p* = 0.0004, *r* = 0.18) and preschool teacher students (*p* = 0.0007, *r* = 0.17). No other pairwise comparisons reached significance after correction.

University PE enjoyment also differed substantially across fields [*H*(3) = 55.83, *p* < 0.001]. Preschool teacher students reported markedly higher university PE enjoyment (*M* = 7.71, SD = 2.39) than special education students (*M* = 6.10, SD = 2.84; *p* < 0.001, *r* = 0.33) and infant and early childhood educator students (*M* = 6.48, SD = 2.82; *p* < 0.001, *r* = 0.25). Social pedagogy students reported the lowest university PE enjoyment (*M* = 6.37, SD = 3.06) and differed significantly from preschool teacher students (*p* < 0.001, *r* = 0.25). Fields also differed significantly in age and work intensity, with preschool teacher and social pedagogy students being older and working more intensively.

Mann–Whitney U tests comparing full-time (*n* = 325) and part-time (*n* = 784) students revealed significant differences on several variables. Part-time students reported higher weekly PA volume (*M* = 2.45 vs. 2.10; *U* = 102,151, *p* < 0.001, *r* = 0.20), higher wellbeing (*M* = 2.77 vs. 2.58; *U* = 101,146, *p* < 0.001, *r* = 0.21), and higher PA importance (*M* = 7.47 vs. 6.93; *U* = 107,849, *p* < 0.001, *r* = 0.15). Full-time students reported more barriers to PA (*M* = 2.35 vs. 2.04; *U* = 150,230, *p* < 0.001, *r* = 0.18) and were markedly younger (*M* = 21.2 vs. 36.5 years) and less likely to be in full-time employment.

### 3.4 Hierarchical regression: predictors of physical activity (Model A)

A hierarchical multiple regression was conducted to examine predictors of weekly PA volume (ordinal, 1–4). The analysis was performed on *N* = 1,086 cases following listwise deletion of missing values on parental education variables. The full results are presented in Table 5.

**Table 5:** Hierarchical regression -predictors of weekly PA volume (Model A).

Predictor	Step 1	SE	Step 2	SE	Step 3	SE	β
	B		B		B		
Age	0.004	0.004	−0.001	0.004	−0.001	0.004	−0.021
Year of study	0.010	0.036	0.009	0.036	−0.001	0.036	−0.001
Study form (Full-time = 1)	−0.157	0.103	−0.145	0.103	−0.124	0.102	−0.073
Financial situation	0.000	0.021	−0.001	0.021	−0.016	0.021	−0.033
Mother’s education	0.009	0.038	0.002	0.038	0.000	0.038	0.001
Father’s education	−0.007	0.045	−0.016	0.044	−0.016	0.044	−0.026
Work intensity**	0.064**	0.024	0.063**	0.024	0.061*	0.024	0.103
Field: infant and early childhood educator	0.122	0.080	0.140	0.080	0.149	0.080	0.083
Field: social pedagogy	0.071	0.101	0.096	0.101	0.090	0.100	0.043
Field: preschool teacher	−0.050	0.083	−0.038	0.083	−0.045	0.084	−0.025
Smoking			0.063	0.070	0.089	0.070	0.047
Alcohol frequency			−0.002	0.050	−0.004	0.050	−0.003
Sleep			−0.069	0.047	−0.084	0.047	−0.062
Healthy eating attention (Q19)			0.084*	0.042	0.027	0.043	0.030
Daily vegetables			0.051	0.031	0.036	0.031	0.082
Daily fruit			−0.004	0.032	−0.010	0.032	−0.020
Fast food			−0.074*	0.034	−0.062	0.034	−0.065
Ready meals			−0.037	0.024	−0.034	0.024	−0.073
Energy drinks			−0.000	0.022	0.008	0.022	0.021
Chronic illness			0.022	0.040	0.039	0.040	0.036
Motivator count					−0.017	0.031	−0.018
Barrier count***					−0.069*	0.028	−0.077
PA importance (Q37)***					0.070***	0.017	0.143
PE enjoyment -secondary school					−0.008	0.012	−0.024
PE enjoyment -university					0.012	0.013	0.034
*R* ^2^	0.040		0.065		0.090		
Δ*R*^2^	0.040		0.025		0.025		
Adj *R*^2^	0.031		0.048		0.068		
F-change	4.44***		2.89**		5.76***		

*N* = 1,086. Reference category for field of study: Special Education. β = standardized coefficient from final model (Step 3). *p < 0.05. **p < 0.01. ***p < 0.001.

Step 1 entered sociodemographic predictors and explained 4.0% of variance in PA volume, F-change (10, 1,075) = 4.44, *p* < 0.001. Among these, work intensity was the only significant predictor (*B* = 0.064, *p* = 0.007). Step 2 added health and lifestyle covariates, yielding a significant increment of Δ*R*^2^ = 0.025, F-change (10, 1,065) = 2.89, *p* = 0.001. Step 3 entered motivation and barrier variables, explaining a further 2.5% of variance, F-change (5, 1,060) = 5.76, *p* < 0.001. The final model accounted for *R*^2^ = 0.090 (Adj R^2^ = 0.068) of variance in PA volume.

In the final model, three predictors reached statistical significance. Perceived importance of exercise (Q37) was the strongest predictor (*β* = 0.143, *B* = 0.070, SE = 0.017, *p* < 0.001), indicating that participants who attributed greater importance to regular PA engaged in more weekly activity. Work intensity also positively predicted PA (*β* = 0.103, *B* = 0.061, SE = 0.024, *p* = 0.010). Barrier count negatively predicted PA (*β* = −0.077, *B* = −0.069, SE = 0.028, *p* = 0.014), such that each additional endorsed barrier was associated with less weekly activity. A sensitivity analysis re-estimating this final model with ordinal logistic (proportional-odds) regression identified the same three significant predictors in the same directions—perceived importance of exercise and work intensity (positive) and barrier count (negative)—indicating that the conclusions were not an artefact of applying OLS to an ordinal outcome.

### 3.5 Hierarchical regression: predictors of wellbeing (Model B)

A four-step hierarchical regression was conducted to predict the wellbeing index (1–4). The same listwise sample was used (*N* = 1,086). Full results are presented in Table 6.

**Table 6:** Hierarchical regression -predictors of wellbeing index (Model B).

Predictor	Step 1	SE	Step 2	SE	Step 3	SE	Step 4	SE	β
	B		B		B		B		
Age	0.004	0.002	0.004	0.002	0.004	0.002	0.003	0.002	0.088
Year of study	0.000	0.020	0.000	0.019	0.000	0.019	−0.010	0.019	−0.027
Study form (nappali = 1)	−0.077	0.058	−0.061	0.056	−0.059	0.056	−0.048	0.055	−0.037
Financial situation***	0.058***	0.012	0.039***	0.012	0.039***	0.012	0.031**	0.012	0.080
Mother’s education	−0.012	0.021	−0.019	0.021	−0.020	0.021	−0.016	0.020	−0.043
Father’s education	0.013	0.025	0.004	0.024	0.004	0.024	0.008	0.024	0.020
Work intensity	0.019	0.013	0.012	0.013	0.010	0.013	0.011	0.013	0.029
Field: infant and early childhood educator ***	0.144***	0.045	0.147***	0.043	0.142***	0.043	0.130**	0.043	0.103
Field: social pedagogy	0.014	0.056	0.053	0.055	0.048	0.055	0.036	0.054	0.022
Field: preschool teacher*	0.109*	0.046	0.105*	0.045	0.105*	0.045	0.065	0.046	0.052
Smoking			0.039	0.037	0.039	0.037	0.037	0.037	0.025
Alcohol frequency			−0.017	0.027	−0.018	0.027	−0.012	0.026	−0.015
Sleep**			0.074**	0.026	0.075**	0.026	0.076**	0.025	0.088
Healthy eating attention (Q19)*			0.076***	0.022	0.065**	0.024	0.052*	0.024	0.073
Daily vegetables***			0.075***	0.012	0.073***	0.012	0.067***	0.012	0.170
Chronic illness*			−0.057**	0.022	−0.057**	0.022	−0.040	0.022	−0.055
PA minutes/week					0.027	0.017	0.023	0.017	0.060
PA frequency index					0.021	0.021	−0.006	0.023	−0.014
Motivator count**							0.048**	0.017	0.089
Barrier count							−0.021	0.015	−0.040
PA importance							−0.001	0.010	−0.005
PE enjoyment -secondary school							0.006	0.007	0.038
PE enjoyment -university**							0.022**	0.007	0.111
*R* ^2^	0.060		0.132		0.135		0.161		
Δ*R*^2^	0.060		0.072		0.003		0.025		
Adj *R*^2^	0.051		0.119		0.121		0.142		
F-change	6.84***		14.79***		2.13		6.40***		

*N* = 1,086. Reference category for field of study: Special Education. β = standardized coefficient from final model (Step 4). *p < 0.05. **p < 0.01. ***p < 0.001.

Step 1 entered sociodemographic variables, explaining 6.0% of variance, F-change (10, 1,075) = 6.84, *p* < 0.001. Financial situation (*β* = 0.080, *p* < 0.001), field of study infant and early childhood educator (*β* = 0.103, *p* = 0.001), and preschool teacher (*β* = 0.052, *p* = 0.019) were significant predictors at this step. Step 2 added health and lifestyle covariates, producing the largest single increment in explained variance: Δ*R*^2^ = 0.072, F-change (6, 1,069) = 14.79, *p* < 0.001, raising the total to *R*^2^ = 0.132. Significant predictors at this step included sleep (*β* = 0.088, *p* = 0.003), healthy eating attention (*β* = 0.073, *p* < 0.001), daily vegetable consumption (*β* = 0.170, *p* < 0.001), and chronic illness (*β* = −0.055, *p* = 0.009).

Step 3 entered PA variables (PA minutes per week and PA frequency index). This increment was not statistically significant (Δ*R*^2^ = 0.003, F-change (2, 1,067) = 2.13, *p* = 0.120), indicating that PA did not explain additional variance in wellbeing beyond what was accounted for by sociodemographic and lifestyle factors. Step 4 entered motivation and barrier variables, adding a significant Δ*R*^2^ = 0.025, F-change (5, 1,062) = 6.40, *p* < 0.001, and raising the final model to *R*^2^ = 0.161 (Adj *R*^2^ = 0.142).

In the final model, seven predictors reached significance. Daily vegetable consumption was the strongest (*β* = 0.170, *p* < 0.001), followed by university PE enjoyment (*β* = 0.111, *p* = 0.002), field of study infant and early childhood educator vs. special education (*β* = 0.103, *p* = 0.003), motivator count (*β* = 0.089, *p* = 0.004), sleep (*β* = 0.088, *p* = 0.003), financial situation (*β* = 0.080, *p* = 0.007), and healthy eating attention (*β* = 0.073, *p* = 0.029). Chronic illness narrowly missed significance in the final model (*B* = −0.040, *p* = 0.065) after being significant in Steps 2 and 3. Neither PA variable reached significance in any step, and neither did barrier count, PA importance, or secondary school PE enjoyment. To address the concern that the reduced dietary specification was data-driven, the wellbeing model was re-estimated with all five eating items entered simultaneously. In this full specification, two unhealthy-eating items—fast food (*β* = 0.08, *p* = 0.016) and ready-meal consumption (*β* = 0.08, *p* = 0.011)—took on positive and statistically significant coefficients, an implausible direction indicative of suppression arising from their intercorrelation with the vegetable and fruit items, whereas daily vegetable consumption remained a positive significant predictor (*β* = 0.15, *p* < 0.001). This pattern confirmed that retaining the full item set produced uninterpretable coefficients and supported the reduced specification reported above.

## 4 Discussion

### 4.1 Lifestyle context explains the physical activity–wellbeing relationship

The most consequential finding of this study is also its least intuitive one: once dietary habits and sleep adequacy were entered into the regression model, weekly physical activity volume ceased to predict subjective wellbeing. The stepwise variance decomposition showed that the lifestyle block comprising vegetable intake, sleep quality, and healthy eating attention accounted for meaningful unique variance in wellbeing, whereas the subsequent entry of PA contributed virtually nothing beyond it. This result does not mean that PA is irrelevant to health in a broader sense; its well-established benefits for cardiovascular outcomes, metabolic risk, and long-term morbidity are not in dispute (1). What it does mean is that, within a simultaneous multivariate model predicting subjective wellbeing in this sample, PA is not an independent predictor of wellbeing in this model. Its apparent bivariate association with wellbeing is better accounted for by shared lifestyle context than by a PA-specific association.

This interpretation is consistent with an emerging literature on health behavior clustering. Similar multidimensional patterns have been identified in Hungarian samples of secondary and university student-athletes, where psychological wellbeing is shaped by a combination of behavioral and psychosocial factors rather than single predictors (21). When healthy behaviors co-occur when individuals who exercise regularly also tend to eat more vegetables, sleep better, and pay greater attention to their diet -isolating the unique contribution of any single behavior becomes methodologically challenging. Alosaimi et al. (22) synthesized evidence from 53 studies and found that clusters combining physical activity, healthy diet, and reduced sedentary behavior were associated with substantially better mental health outcomes than any single behavior studied in isolation, with the combined profile accounting for effects that exceeded what individual predictors would suggest additively. Wickham et al. (23), using a comparable hierarchical regression design in young adults, similarly found that sleep quality was the dominant predictor of both depressive symptoms and flourishing once physical activity and diet were modelled jointly a structural parallel to our own findings. This convergence across different samples and national contexts suggests that the attenuation of PA’s predictive role under lifestyle controls is not a measurement artefact but a substantively important regularity.

For practitioners and curriculum designers working with pre-service helping professionals, this finding carries a specific message. Interventions framed narrowly around increasing PA volume step counts, gym attendance, structured exercise sessions may produce limited wellbeing returns if dietary quality and sleep remain poor. A lifestyle-oriented framing, targeting the coherent adoption of multiple health behaviors simultaneously, is more consistent with what the current and converging data suggest. In practical terms, this favors integrated university health-promotion programs that simultaneously address physical activity, nutrition, and sleep, rather than single-behavior campaigns.

### 4.2 Vegetable consumption and dietary attention as wellbeing predictors

Daily vegetable consumption emerged as the single strongest predictor of wellbeing in the final model, a finding that adds specificity to the broader diet-mental health association documented in the literature. Solomou et al. (14) concluded in their systematic review that unhealthy dietary patterns in university students were consistently associated with poorer mental health outcomes, but noted considerable heterogeneity in how diet was operationalized across studies. Our results suggest that a single behaviorally concrete indicator daily vegetable consumption as a yes/no habit carries predictive power comparable to, or exceeding, more elaborate dietary indices. This is practically meaningful: it implies that a straightforward, easily measurable eating behavior can serve as both an indicator and a potential intervention target. At the same time, daily vegetable consumption may function less as an isolated nutritional cause than as a marker of a broader health-conscious lifestyle, co-occurring with other, unmeasured health-supporting behaviors; its strong predictive value should accordingly be read as partly indicative of this wider pattern rather than as an isolated dietary effect.

The additional independent contribution of healthy eating attention operationalized as the degree to which respondents reported consciously managing their diet extends this finding in an important direction. Dietary attention is not simply a proxy for diet quality; it captures an orientational, self-regulatory dimension of food behavior. Students who report attending to their eating are, in effect, exercising a form of intentional self-care that may itself be wellbeing-relevant, independent of whatever nutritional benefits the behavior produces. This is consistent with research on autonomous regulation in health behavior: individuals who endorse health-related activities because they value them and find them personally meaningful show better psychological outcomes than those engaging in identical behaviors for external reasons (9, 10). In this sense, dietary attention may function as a marker of identified or integrated regulation applied to the eating domain a proxy for self-determined health orientation more broadly.

### 4.3 Sleep as a wellbeing predictor

Sleep adequacy emerged as a significant predictor of wellbeing independently of both dietary and PA variables (23). This is consistent with a substantial body of evidence documenting the psychological costs of poor sleep in university student populations (17, 18), as well as findings from Hungarian population-based research demonstrating associations between sleep duration and subjective wellbeing (24). What our data add to this literature is a demonstration that sleep retains independent predictive value even when simultaneously modelled against diet quality and PA a relatively rare analytical configuration in the student wellbeing literature, where sleep, diet, and PA are more commonly studied in separate research traditions than modelled jointly. The fact that all three survived as significant predictors in the final model, despite their expected intercorrelations, supports the view that each contributes a distinct pathway to wellbeing rather than serving as interchangeable proxies for a single latent health orientation (16).

For the specific population studied here predominantly female, partly employed, many combining full-time work with part-time study sleep pressure is plausibly considerable. Time-budget constraints created by simultaneously managing study deadlines, paid employment, and family or household responsibilities may compress the opportunities for adequate sleep in ways that differ from those of traditional full-time students. Future research with this population would benefit from moving beyond self-reported sleep adequacy to examine sleep duration and quality directly, and from exploring the specific sources of sleep disruption that are most salient for working adult learners in helping professions.

### 4.4 PA importance and enjoyment as motivational architecture

Among the predictors of PA itself, perceived importance of physical activity proved the strongest, outperforming structural variables including barrier count and work intensity. This finding maps straightforwardly onto the self-determination theory framework (9): valuing a behavior the hallmark of identified regulation is among the most reliable predictors of whether people actually perform it (25). In the present sample, where direct assessment of motivational regulation was constrained by the survey instrument, PA importance serves as a functional proxy for the identified end of the motivational continuum. Participants who attributed high importance to physical activity were more physically active regardless of other contextual factors, suggesting that value-internalization, rather than opportunity or access, is the primary lever for this population.

The independent predictive contribution of PE enjoyment at university deserves particular attention. Although the study was cross-sectional and causality cannot be established, the association between positive PE experiences during university education and current PA volume is theoretically coherent and empirically supported across contexts. Teixeira et al. (26) demonstrated that exercise enjoyment is a robust predictor of exercise habit formation and intention to continue exercising, with effects operating through both direct and attitude-mediated paths. Guan et al. (27) found that PE enjoyment predicted PA engagement frequency in adolescents through its interaction with perceived competence a relationship that is plausibly generalizable to young adults. Yan et al. (28) showed that childhood memories of enjoyment or non-enjoyment during PE were associated with adult PA intentions and sedentary behavior. Taken together, these findings suggest that the quality of institutional PE experiences shapes a motivational disposition toward physical activity that persists beyond the formal educational context. For students enrolled in programs that explicitly include movement-related content as several of the programs in our sample do the affective quality of those experiences may matter as much as their content.

The positive contribution of motivator count to PA is consistent with research showing that motivational breadth having multiple reasons to be active supports more robust and sustained engagement than reliance on any single motivating factor (25). Conversely, the negative effect of barrier count confirms the established primacy of time scarcity and competing demands in suppressing PA among students (4). What is notable here is that both motivational and barrier variables predicted PA simultaneously and independently, suggesting that intervention efforts need to operate on both sides of this push-pull dynamic: strengthening reasons to be active is not sufficient if structural barriers remain unaddressed, and removing barriers alone does not guarantee engagement if motivational foundations are weak.

### 4.5 The part-time paradox: higher PA and wellbeing in working adult students

A notable counterintuitive pattern in the data concerns study form. Part-time students, who are substantially older and who more frequently combine full-time employment with their studies, reported higher PA levels and better subjective wellbeing than their full-time counterparts. This is the inverse of what might be predicted from a resource-depletion perspective, in which the combined demands of employment and study would be expected to crowd out both health behaviors and wellbeing. Several non-exclusive explanations deserve consideration.

First, part-time students in the Hungarian higher education system are typically working adults who have chosen to re-enter education alongside existing employment and life commitments. This volitional orientation returning to study by choice may itself be associated with higher levels of self-determination and life purpose, both of which predict subjective wellbeing independently of lifestyle factors. Second, employment provides not only time constraints but also meaningful social roles, routine structure, and financial stability, all of which can support wellbeing. Third, part-time students are older on average, and research suggests that age-related increases in life experience, emotional regulation capacity, and goal clarity may independently support both wellbeing and health behavior self-management. Finally, the employment itself frequently involves physical or semi-physical work a form of incidental PA that would inflate reported activity levels relative to sedentary full-time students who spend extended periods studying at desks or in front of screens. The work intensity variable in the PA model supports this interpretation: higher work intensity was positively associated with total weekly PA, implying that occupational physical activity contributes meaningfully to the overall PA profile of these students.

This pattern has practical implications for health promotion targeting in this population. Standard public health PA messaging, which addresses leisure-time sport and structured exercise, may be poorly calibrated for working adult students whose primary PA comes from occupational activity. Surveillance instruments and intervention designs need to account for occupational and commuting PA to avoid systematically misrepresenting the health profiles of non-traditional student populations.

### 4.6 Program-level wellbeing differences: the case of special education students

Students enrolled in the special education program reported lower subjective wellbeing than those in infant and early childhood education and pre-school education programs, a difference that persisted in exploratory analyses and warrants interpretive consideration.

The professional context these students are preparing to enter is well-documented as one of the most emotionally demanding in the helping professions. Special educators are expected to form intense, sustained relationships with children who may present with complex, challenging, or distressing needs; they carry heavy individual advocacy and documentation responsibilities; and they frequently operate within underresourced institutional settings (29, 30). Research on in-service special educators consistently documents elevated rates of emotional exhaustion, burnout, and psychological distress relative to other teaching populations (29, 31). What the present data suggest is that an anticipatory dimension of this demand may already be visible at the pre-service stage: students who are beginning to build a professional identity centered on intensive relational work with vulnerable populations may already be engaging in a form of emotional labor that taxes their wellbeing resources even before formal employment begins.

This interpretation should be treated as provisional given the cross-sectional and exploratory nature of the program-level comparison. It is possible that program-level differences in academic load, placement demands, or student selection effects account for some of the observed variation. Nevertheless, the finding aligns with growing recognition that professional socialization in high-demand helping professions begins during training, and that wellbeing support for pre-service special educators may need to be designed with the specific emotional demands of the role in mind, rather than delivered through generic student wellness programs.

### 4.7 Limitations

Several limitations must be considered when interpreting the findings of this study. The most fundamental is its cross-sectional design. All measurements were collected at a single time point, which means that the direction of the observed associations cannot be determined. The relationship between sleep and wellbeing, for example, is plausibly bidirectional: poor wellbeing may disrupt sleep as readily as poor sleep impairs wellbeing. The same applies to dietary behavior and to PA. Establishing causal ordering requires longitudinal data, ideally with repeated measurement across the academic year or across the transition into professional practice.

The sample was drawn from a single university the University of Debrecen, Hungary and from a specific cluster of programs within it. Although the sample is reasonably large and the programs studied are structurally representative of helping-profession preparation in Hungary, the findings cannot be generalized beyond this institutional and national context without replication. The sample is also strongly female-dominated, which reflects the actual gender composition of these programs in Hungary but limits comparability with studies conducted in more gender-balanced student populations. Accordingly, the findings should not be assumed to generalize to male students or to more gender-balanced cohorts, and the external validity of the results is correspondingly constrained.

All variables were measured through self-report, which introduces the risk of social desirability bias and retrospective inaccuracy, particularly for behavioral variables such as physical activity, vegetable consumption, and sleep. Physical activity was assessed using a single item capturing weekly sport time, categorized into ordinal bands. This is a pragmatic measure that is easily administered in survey research but lacks the temporal precision and domain coverage of validated instruments such as the International Physical Activity Questionnaire (IPAQ). In particular, the item does not capture non-sport physical activity, including active commuting, occupational physical activity, and household tasks forms that are likely to constitute a substantial portion of the PA profile of employed part-time students. The finding that work intensity positively predicts PA volume is consistent with this gap: occupational activity is contributing to PA levels in ways the sport-time item alone does not capture. This measurement limitation bears directly on the central finding that PA volume did not independently predict wellbeing: a more comprehensive, validated PA instrument might capture activity dimensions more closely linked to wellbeing, and the null result should therefore be read as specific to the sport-time measure used here rather than as evidence against any PA–wellbeing association. The regression models also explained only a modest share of variance in the outcomes (*R*^2^ = 0.09 for weekly PA volume and *R*^2^ = 0.16 for wellbeing), leaving most variance unexplained; important determinants were therefore not captured by the present predictors—plausibly including personality, social support, academic and financial stress, clinical mental-health status, and objectively measured activity, diet, and sleep—so the findings identify a limited set of associated factors rather than a comprehensive explanatory model. Finally, the selection of dietary predictors for the wellbeing model was partly data-driven, guided by collinearity and suppression diagnostics; although this was done to avoid uninterpretable coefficients and is fully reported, such data-driven specification carries a risk of overfitting and should be confirmed in independent samples.

The motivational assessment was similarly constrained. The survey included items on PA importance, PE enjoyment, motivator count, and barrier count, which served as proxies for motivational constructs within a self-determination theory framework. However, a full operationalization of SDT’s motivational continuum distinguishing intrinsic motivation, identified regulation, introjected regulation, and external regulation through validated scales was not achieved due to a response scale ambiguity in the original instrument. The motivational findings should therefore be interpreted within the limits of these proxy measures rather than as direct tests of SDT propositions.

The wellbeing outcome was assessed using a five-item index adapted from the WHO-5 Wellbeing Index with a modified four-point response scale. Because this response format departs from the original six-point WHO-5, the resulting scores are not directly comparable with the validated instrument, and the adapted version was not separately validated beyond the internal-consistency analysis reported above. Multidimensional wellbeing instruments capturing hedonic and eudaimonic components, positive and negative affect, and domain-specific satisfaction would have provided a richer and more differentiated picture of student wellbeing in this population.

Finally, the program-level comparison between study programs (special education, infant and early childhood education, preschool education, social education) was exploratory in nature. The cell sizes for some programs are small, and the observed differences should not be over-interpreted. Replications with larger per-program samples and with control for potential confounds including academic year, placement experience, and socioeconomic background would be required before program level conclusions could be drawn with confidence.

## 5 Conclusion

This study examined physical activity levels, subjective wellbeing, and their predictors in a sample of pre-service students enrolled in helping-profession programs at a Hungarian university. Three substantive conclusions can be drawn.

First, physical activity volume does not independently predict subjective wellbeing once dietary habits and sleep adequacy are entered into the same model. The apparent association between PA and wellbeing observed at the bivariate level is better explained by shared lifestyle context a coherent pattern of health-supporting behaviors than by a PA-specific association with psychological wellbeing. Vegetable consumption and sleep adequacy were the dominant wellbeing predictors, with dietary attention making an additional independent contribution. This finding challenges the common assumption that targeting PA is equivalent to targeting wellbeing, and calls for health promotion approaches in this population to be framed around lifestyle coherence rather than PA volume alone.

Second, in the present model, PA behavior was associated more strongly with motivational and structural factors than with demographic characteristics. Perceived importance of physical activity was the strongest predictor, consistent with self-determination theory’s proposition that value-internalization may be an important motivational correlate of sustained health behavior. Positive experiences of physical education.

Third, the study reveals two patterns that are specific to its population and context and that enrich the broader literature. Working adult (Part-time) students reported higher PA and better wellbeing than their full-time peers despite greater competing demands, a pattern that may partly reflect occupational physical activity, role-derived purpose, and age-related advantages. Students in the special education program reported lower wellbeing than those in other programs, a difference that is theoretically consistent with the known emotional demands of the professional role they are preparing to enter, and that may warrant tailored wellbeing support at the pre-service stage.

Together, these findings contribute to a more nuanced understanding of student health by demonstrating that the relationship between PA and wellbeing is conditional on the broader lifestyle context in which PA occurs, by documenting the relevance of motivational quality for PA engagement in a population that has received minimal empirical attention, and by highlighting structural features of Hungarian higher education the prevalence of adult working learners, the emotionally intensive nature of helping-profession training that shape student health in ways that generalized research models do not capture. Future research should extend these findings through longitudinal designs, validated multi-domain instruments, and comparative studies across institutional and national contexts.

## Data Availability

The datasets generated and/or analysed during the current study are available from the corresponding author upon reasonable request.
